# Risk of Gynecological Cancers in Women With Polycystic Ovary Syndrome and the Pathophysiology of Association

**DOI:** 10.7759/cureus.37266

**Published:** 2023-04-07

**Authors:** Chaitra Shetty, Syed Muhammad Hannan Ali Rizvi, Joudi Sharaf, Kerry-Ann D Williams, Maha Tariq, Maitri V Acharekar, Sara Elena Guerrero Saldivia, Sumedha N Unnikrishnan, Yeny Y Chavarria, Adebisi O Akindele, Ana Paula C Jalkh, Aziza K Eastmond, Pousette Hamid

**Affiliations:** 1 Research, California Institute of Behavioral Neurosciences and Psychology, Fairfield, USA

**Keywords:** pathophysiology of cancer in pcos, pcos and cancer risk, pcos and vulval cancer, pcos and cervical cancer, pcos and breast cancer, pcos and endometrial cancer, pcos and ovarian cancer, polycystic ovarian syndrome and gynecological cancers, gynecological cancers, pcos

## Abstract

Polycystic ovary syndrome (PCOS) is an endocrine disorder increasingly affecting women in the reproductive age group. The women usually present with menstruation irregularities, hirsutism, weight gain, and acne. There has been ongoing research about the increased risk of gynecological cancers in women with polycystic ovary syndrome compared to those without it. This review aimed to understand the risk of gynecological cancers, endometrial, ovarian, and breast cancer in PCOS, and to study in detail the underlying mechanisms involved. We searched PubMed and Google Scholar databases for studies and selected 10 articles from a total of 19,388 relevant articles. We found an increased risk of endometrial cancer in women with PCOS whereas the risk of ovarian and breast cancer was not increased. A recent study has even reported a reduced risk of ovarian cancer in genetically predicted PCOS. In understanding various medical conditions possibly leading to cancer in these women we found that hyperandrogenism, hyperinsulinemia, unopposed estrogen action, chronic inflammation, and dyslipidemia were major contributors. There is a need for more large-scale cohort studies which will take into consideration other factors leading to cancers in women with PCOS, such as smoking, alcohol, and family history, to substantiate the significance of these associations further. The interventions used to treat PCOS might also affect the risk of cancer and require further probing. This review is an attempt to analyze the risk of cancers of the reproductive system in females with PCOS in coherence with understanding the mechanisms leading to the respective cancers.

## Introduction and background

Polycystic ovary syndrome (PCOS) is a complex endocrine disorder presenting with oligomenorrhea, hyperandrogenism, and polycystic ovaries [1]. Many metabolic abnormalities, such as dyslipidemia, insulin resistance, and type II diabetes, are associated with PCOS and PCOS is also one of the most common causes of reduced fertility. PCOS alters the hormonal and metabolic environment in women which leads to an increased risk of certain cancers [2,3]. The diagnosis of PCOS is difficult and commonly delayed due to its heterogeneous nature [4]. PCOS is diagnosed with the help of a few different criteria. According to the National Institutes of Health in 1990, PCOS is confirmed if a woman presents with oligo-ovulation (OA), clinical or biochemical hyperandrogenism (HA), and an ultrasound showing polycystic ovarian morphology (PCOM) [5]. The presence of any two of OA, HA, and PCOM is sufficient to diagnose PCOS according to the 2003 Rotterdam criteria [6]. The Androgen Excess and Polycystic Ovary Syndrome (AE-PCOS) Society criteria are the presence of hyperandrogenism and oligo-ovulation or polycystic ovarian morphology or both [7]. The Rotterdam criteria are accepted worldwide now. They describe four PCOS phenotypes in adult women which can be described as follows: (A) OA + HA + PCOM, (B) OA + HA, (C) HA + PCOM, and (D) OA + PCOM [6]. The 2018 International PCOS evidence-based guideline recommends and endorses the Rotterdam criteria. A team of multidisciplinary clinicians and researchers across 37 societies from 71 countries, co-developed the criteria with consumer engagement and they were based on unprecedented evidence synthesis and best practice methods [8].

**Figure 1:**
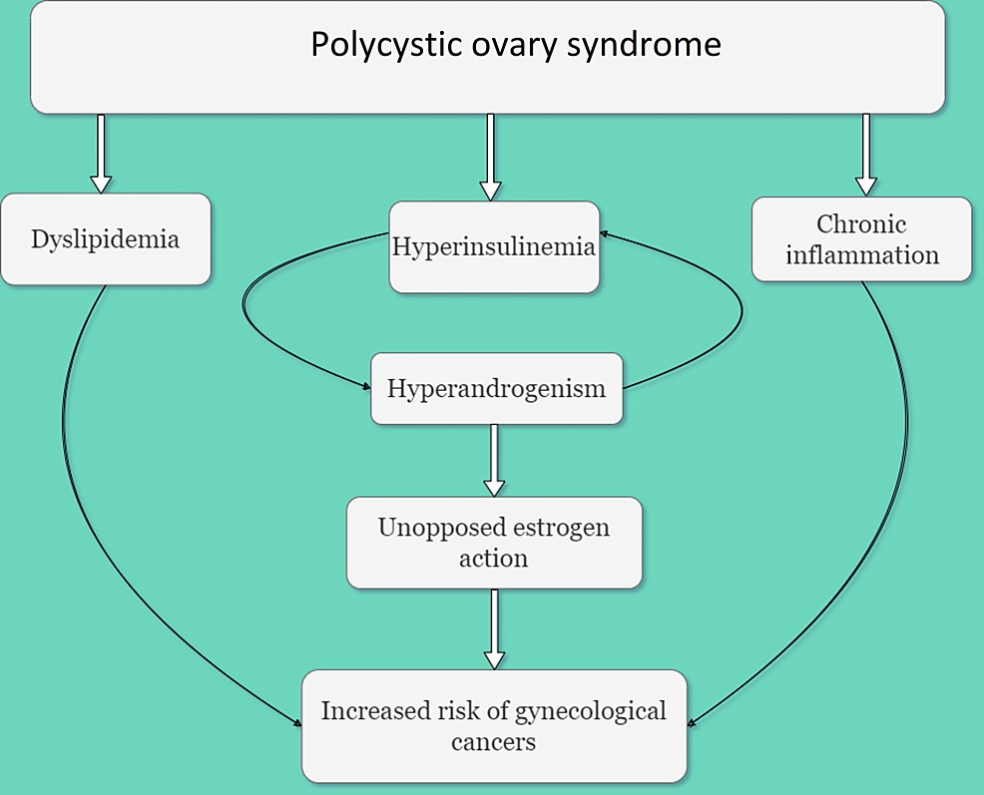
Processes involved in the pathogenesis of cancer in polycystic ovary syndrome. The image is created by the authors of this study by using "draw.io."

Several medical conditions, such as dyslipidemia, hyperinsulinemia, and chronic inflammation, are associated with PCOS [9]. Chronic anovulation can lead to endometrial hyperplasia because of prolonged exposure to unopposed estrogen, which can progress to carcinoma. Women with PCOS suffer from ovulation irregularities which results in infrequent or absent endometrial shedding and thus they are at a higher risk of undergoing endometrial hyperplasia and developing endometrial carcinoma [10]. The growth of breast and ovarian carcinoma which are hormone-sensitive tumors can be explained by the sustained increase in concentrations of serum estrogen (Figure 1) [11]. The correlation between ovarian carcinoma and PCOS is not fully known yet and studies that have looked into it have determined that the risk of ovarian cancer in women with PCOS is not greatly elevated [12]. The Ovarian Cancer Association Consortium (OCAC) recently found a lower risk of invasive ovarian cancer in women with self-identified PCOS. However, considering the limits of self-reported PCOS, the validity of these findings is debatable [13]. There hasn't been enough research conducted to understand if and how there is an association between PCOS, and cancers of the vagina, vulva, and cervix. There also doesn't seem to be any plausible mechanism in place explaining the correlation [11].

Women with PCOS have three times more probability of developing endometrial carcinoma as compared to those without PCOS. This corresponds to a 9% lifetime risk of endometrial carcinoma among Caucasian women with PCOS versus a 3% lifetime risk in women without PCOS. Although the majority of women with PCOS (91%) will not develop endometrial cancer, they are at a higher risk [14]. PCOS, on the other hand, was not linked to ovarian or breast cancer [15]. In the multivariable-adjusted study, neither infertility nor related diseases were linked to an increased risk of breast cancer [16].

It is observed that the different medical conditions occurring as a result of PCOS in females could be leading to cancers of the reproductive system. Even though there's substantial information about the risk of gynecological cancers in PCOS, there seemed to be a gap in the availability of literature explaining the risk of each gynecological cancer and the specific medical condition in PCOS leading to it. This review aimed to further elaborate on the link between the occurrence of these gynecological cancers in these women and PCOS. We have attempted to bring together the literature on different gynecological cancers occurring in females with PCOS and hope to understand the causation and pathophysiology of these cancers. At the same time, we hope to highlight the scope of research in this area.

## Review

Methods

**Figure 2:**
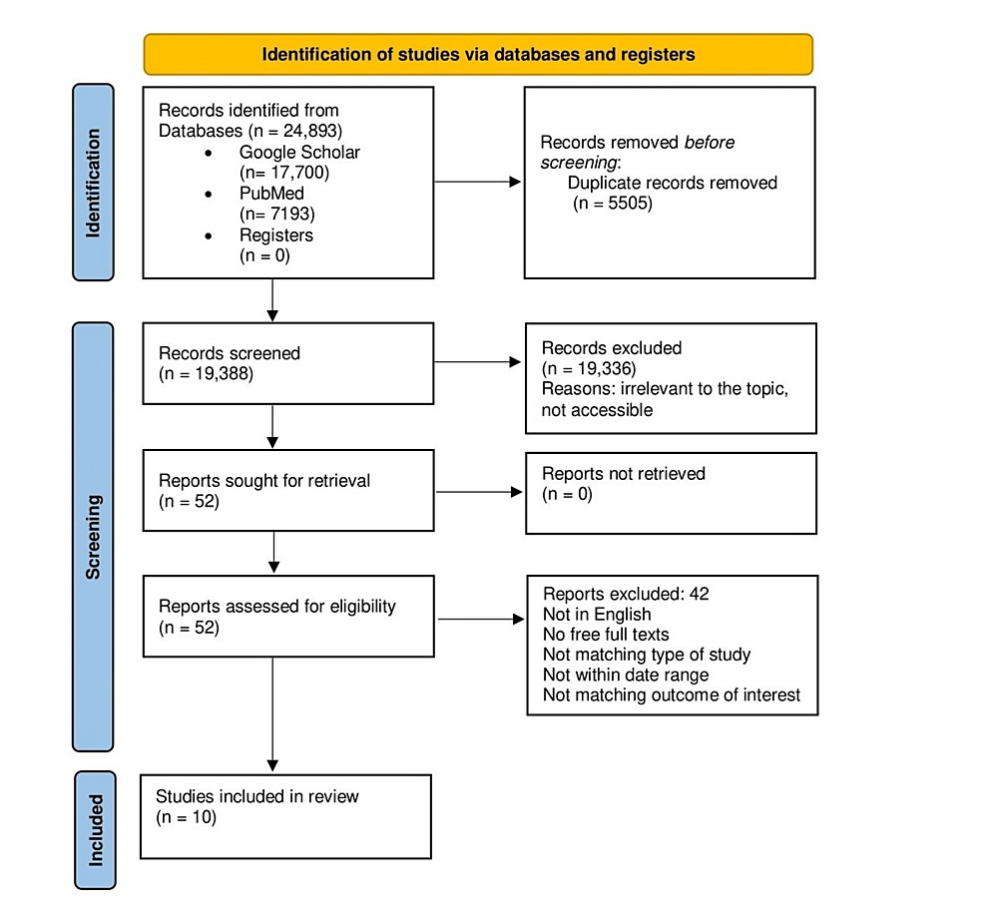
PRISMA flow diagram showing the search strategy used for article selection. PRISMA: Preferred Reporting Items for Systematic Reviews and Meta-Analyses

We searched the following electronic bibliographic databases; PubMed and Google Scholar to identify publications about the association between polycystic ovary syndrome and gynecological cancers. We used the following keywords: "polycystic ovary syndrome and gynecological cancers, PCOS and ovarian cancer, PCOS and endometrial cancer, PCOS and breast cancer, PCOS and cervical cancer, PCOS and vulval cancer." The search in Google Scholar resulted in 17,700 articles. We selected a total of 12 relevant articles from them. The PubMed search resulted in a total of 7,193 articles, out of which a total of 40 articles were selected. For this traditional review, the following inclusion criteria were applied: (a) publications in the English language only, (b) full free texts and abstracts, (c) no grey literature included, (d) date range: 2010-2022, (e) observational studies, meta-analysis, and systematic review included, (f) outcome studied was gynecological cancer. We excluded (a) outcomes with other cancers, (b) languages other than English, (c) studies before 2010, and (d) traditional reviews. The duplicates were removed manually with the help of an Excel sheet. After screening articles for titles and abstracts 52 articles that were relevant to our research question were identified. A total of 10 studies from 52 articles were selected for review, after applying the aforementioned criteria. Nine of the selected studies were full free texts and one was an abstract of an observational study. Cross-referencing the source material was done wherever required and this led to the additional articles being referenced. The PRISMA diagram below shows the flow of the article selection process (Figure 2) [17].

Discussion

Endometrial Cancer

**Figure 3:**
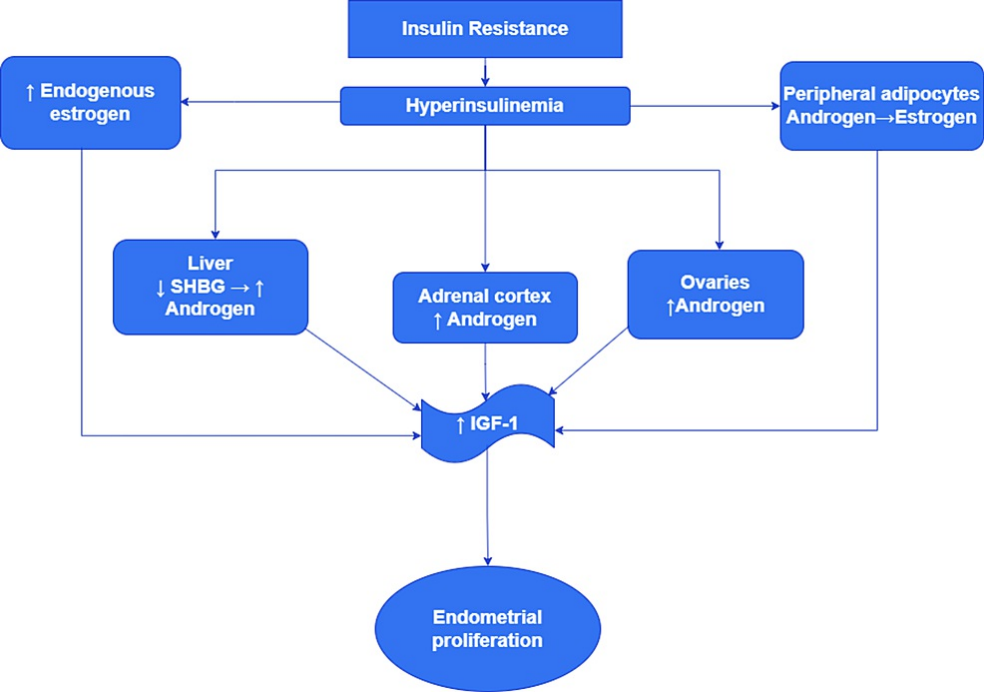
Steps in the hyperinsulinemia cascade leading to endometrial proliferation. SHBG: sex hormone-binding globulin; IGF-1: insulin-like growth factor-1 The image is created by the authors of this study by using "draw.io."

Obesity, nulliparity, age > 50, infertility, hypertension, diabetes, chronic anovulation, and unopposed estrogen supplementation are all significant risk factors for endometrial carcinoma in women with PCOS [10]. Hyperinsulinemia increases adrenal and ovarian androgen synthesis, lowers hepatic sex hormone-binding globulin formation, and increases endogenous estrogen production from progesterone. Additionally, aromatase enzymes which are present in visceral adipocytes convert androgens to estrogens. In anovulatory women, the average progesterone levels are significantly decreased. Insulin, androgens, and estrogens all stimulate mitotic activity through increasing insulin-like growth factors. These modifications may raise the risk of endometrial hyperplasia and endometrial carcinoma by stimulating endometrial proliferation and mutagenesis potential (Figure 3) [18]. We have included two systematic reviews which have 11 studies, three meta-analyses, and five individual studies. In addition to that, we have assessed six individual cohort studies examining the association between PCOS and endometrial cancer for this review.

A meta-analysis done in 2012 by Houla et al. explored data from five studies with a total of 4,605 females showing that 88 had PCOS and 4,517 didn't have PCOS [14]. In the PCOS group, endometrial cancer was diagnosed in 47 females. Whereas, in the non-PCOS group 773 females were diagnosed with it. The odds of developing endometrial cancer were about three times greater in women with PCOS than in women without PCOS, according to the aggregated data, with the confidence intervals (CI) > 1 (OR: 2.89, 95% CI: 1.52-5.48). In Caucasian women with PCOS, this amounts to a 9% lifetime risk of endometrial cancer, compared to 3% in women without PCOS. This is the first study to show a clear relationship between PCOS and endometrial cancer in a systematic review and meta-analysis. This research adds to the growing body of evidence supporting the relationship between PCOS and endometrial cancer. This finding is crucial since it can be used to detect cancer early on by implementing a screening program. Early detection may improve outcomes as endometrial cancer has a five-year survival rate of 86% [14]. A Thai cross-sectional study of 52 patients concluded that the percentage of abnormal endometrial pathology in PCOS patients presenting with abnormal menstrual patterns was 19.2% (17.3% for endometrial hyperplasia and 1.9% for endometrial cancer) [18]. When compared to other research, the percentage of endometrial hyperplasia was 17.3%, which is significantly lower. PCOS patients with amenorrhea may have an increased prevalence of endometrial hyperplasia than PCOS individuals with an irregular menstrual pattern. The percentage of endometrial cancer, on the other hand, was identical to prior reports [19].

A retrospective observational transactional study done in Denmark in 2012 involving 963 women showed that six of them had simple hyperplasia without atypia, three had complex hyperplasia without atypia, and one had complex atypical hyperplasia [18]. Over nine years, only one woman experienced more than one incidence of endometrial hyperplasia, i.e., three. Otherwise, each example had only one instance of endometrial hyperplasia. One lady (0.1%) developed endometrial cancer, which was type 1 low-grade endometrial adenocarcinoma, International Federation of Gynecology and Obstetrics (FIGO) stage IA, with prior irregular proliferation and complex hyperplasia, which is similar to the general population and lower than other studies' findings [18]. The study showed an almost similar prevalence of endometrial cancer in the PCOS population as in the general population, owing to the increased awareness about the importance of regular endometrial shedding imparted to the susceptible population in the country in the form of hormonal treatments. This implies that women with undiagnosed PCOS and/or hirsutism might have a higher risk of endometrial hyperplasia and endometrial cancer than those found in the present study [18]. The systematic review by Harris and Terry identified 11 individual studies and three meta-analyses on the associations between PCOS and endometrial cancer and clearly stated the importance of considering body mass index (BMI) and that BMI may be both a mediator and confounder of the PCOS and cancer associations, making it difficult to characterize a BMI-independent PCOS association [1]. PCOS diagnostic criteria, etiologic heterogeneity of cancer subtypes, confounding and mediating factors, menopausal status, co-morbid conditions, and treatment options that may also influence cancer risk are all factors to consider when looking at the links between PCOS and endometrial, ovarian, and breast cancer. Barry et al. in their systematic review included a heterogeneous group of studies basis their diagnostic criteria for PCOS [12]. There is evidence of heterogeneity in this study due to the PCOS phenotypes variation because of the use of different diagnostic criteria, patients belonging to different ethnicities, and the size of the cohort. A random-effects model was used to mitigate the effects of heterogeneity, where appropriate. Some of the studies' CIs crossed zero, indicating that no significant risk could be calculated, though endometrial cancer was found to have considerable risk. The other equally important finding reached from our comprehensive review is that the current evidence is far from robust, and that differences in PCOS diagnostic criteria, associated risk factors (especially obesity), and selection bias may have resulted in an exaggeration of the elevated risk [11].

In a large Taiwan cohort research conducted by Ding et al., more than 8,155 Taiwanese women with PCOS were shown to have a 17-fold greater risk of endometrial cancer than women without PCOS [15]. Similarly, Lundberg et al. concluded that infertile women have a greater risk of endometrial cancer than fertile women, with PCOS as the underlying cause [16]. Shen et al. added to the growing body of evidence that PCOS patients have an increased risk of uterine cancer, the most frequent form of which is endometrial cancer [20]. Cancer was discovered in 279 PCOS women. They discovered a nearly four-fold increased risk of endometrial cancer, with type 1 cases accounting for the vast majority of cases [21].

Ovarian Cancer 

PCOS raises the chances of ovarian cancer due to elevated androgen exposure, owing to the existence of androgen receptors on both normal and benign ovarian cells [1]. An earlier meta-analysis by Chittenden et al. in 2009 found a doubling of the incidence of ovarian cancer in women with PCOS compared to controls (OR: 2.52, 95% CI, 1.08-5.89) [11]. Meta-analysis could not be performed since the results were from a single study with ovarian cancer as the outcome. It was discovered that women with PCOS are twice as likely as those without the disorder to develop ovarian cancer [11]. According to a systematic review by Barry et al., the increased risk in women with PCOS was not significant in the three studies that revealed ovarian cancer, but the OR was greater and significant in one study of women aged > 54 years [12]. High BMI is a known risk factor for endometrial and breast cancer, with some evidence of a link with ovarian cancer. However, this elevated risk may not apply to invasive serous malignancies [22]. As a result, our discovery of a higher risk of endometrial cancer is due, at least in part, to the higher frequency of obesity in women with PCOS. In support of this idea, we discovered that in studies where BMI was similar in the PCOS and non-PCOS groups, the risk of ovarian cancer was somewhat reduced. The other extremely valuable conclusion to be derived from this systematic review is that the existing data is far from robust, implying that differences in PCOS diagnostic criteria, risk factors associated (particularly obesity), and selection bias may have contributed to an overestimation of the increased risk [12]. Shen et al. in their systematic review reaffirmed the findings of Barry et al. that the risk of ovarian cancer was not significantly increased in women with PCOS [12,20]. Harris and Terry in 2016 in their systematic review highlighted the association between the serous borderline subtype of ovarian cancer and PCOS but at the same time, it postulated the correlation between that and evidence of BMI > 25 in those females [1]. Compared to the general population, women with PCOS had a non-significant risk of ovarian cancer, according to Gottschau et al. [21]. In Taiwanese cohort research, Ding et al. found no link between PCOS and ovarian cancer [15]. Thus, patients with PCOS are typically treated with oral contraceptives according to the guidelines that suggest that oral contraceptives may counteract the potential cancer-promoting effects of PCOS.

Infertile women had a higher incidence risk of ovarian cancer, according to a recent population-based cohort study by Lundberg et al. [16]. They also discovered that women diagnosed with ovulatory abnormalities had a greater risk of ovarian cancer, which appeared to be limited to nulliparous premenopausal women. Although estimates were based on small numbers, women diagnosed with both infertility and ovulatory disturbances did not have a markedly higher risk of ovarian cancer. They discovered an increased risk of ovarian cancer in women with endometriosis [16]. Surprisingly, another recent Mendelian randomization study found an inverse relationship between genetically predicted PCOS and the risk of invasive ovarian cancer, with the endometrioid histotype showing the strongest inverse association. And, after controlling for BMI (potential confounder), oral contraceptive use (potential mediator), and parity (potential mediator), the risk of ovarian cancer did not change significantly [13].

Breast Cancer

Breast cancer is a heterogeneous condition. The incidence, clinical features, and prognosis of breast cancer vary markedly based on ethnicity and race [23]. Breast cancer is linked to reproductive factors, such as early menarche before the age of 12 years, late menopause after the age of 55 years, nulliparity, late age at first birth, miscarriages before the first full-term pregnancy, infertility, and hormone use, according to several epidemiological studies. Breast cancer is also linked to environmental variables, such as high socioeconomic status, obesity, certain eating habits, alcohol use, poor physical activity, and ionizing radiation (therapeutic uses) [24-30]. We included six studies that examined the association between PCOS and breast cancer. In a comprehensive study, Harris and Terry found that women who reported infertility owing to ovulatory problems had no increased risk of breast cancer [1]. Compared to women with no documented infertility, women with ovulatory problems who were treated for infertility had a much-decreased risk [31]. Because of anovulatory cycles in PCOS and other ovulatory diseases, the lifetime exposure of breast cells to luteal phase hormones, such as estrogen and progesterone, is inadvertently reduced. In their systematic review, Barry et al. found that the risk of breast cancer in PCOS women was not statistically different from control women overall, or in the subgroup analysis of two studies of younger women with breast cancer [12]. In the multivariable-adjusted study, neither infertility nor associated disorders were linked to a greater risk of breast cancer [16]. In a 2018 population-based cohort study in Taiwan, no link was found between PCOS and ovarian cancer (adjusted hazard ratio {aHR}: 1.64, 95% CI: 0.63-4.27) or breast cancer (aHR: 0.98, 95% CI: 0.58-1.65) [15]. Women with PCOS did not have a substantially lower adjusted hazard ratio (aHR) for breast cancer than patients without PCOS [15]. According to a 2014 retrospective cohort study, the aHR for breast cancer development was similarly greater than that of control individuals (aHR: 1.98, 95% CI: 1.04-3.77) [19]. The Monte Carlo technique, on the other hand, did not result in a statistically significant rise in the mean adjusted HR of breast cancer. This conclusion backed the prior research suggesting that women with PCOS do not have a higher risk of breast cancer.

In Table 1, below, characteristics of the 10 studies included in the review to assess the risk of gynecological cancers in women with polycystic ovarian syndrome are summarized.

**Table 1:** Characteristics of the 10 studies included in the review to assess the risk of gynecological cancers in women with polycystic ovary syndrome. PCOS: polycystic ovary syndrome

Study	Journal	Study type	Year	No. of patients/studies	Aim of the study	Conclusion
Lundberget al. [16]	European Journal of Epidemiology	Population-based cohort study	2019	2,882,847 women	To understand the association between infertility and the risk of ovarian, endometrial, and breast cancer and whether it could be explained by ovulatory disturbances, endometriosis, or nulliparity	Infertility was associated with a higher incidence rate of ovarian and endometrial cancer but not of breast cancer
Harris et al. [13]	International Journal of Epidemiology	Mendelian randomization study	2019	22,406 with invasive disease, 3,103 with borderline disease, and 40,941 controls	To assess the association between genetically predicted PCOS and ovarian cancer	There is an inverse association between genetically predicted PCOS and invasive ovarian cancer risk
Ding et al. [15]	Medicine	Population-based cohort study	2018	8,155 patients with PCOS and the comparison cohort consisted of 32,620 matched patients without PCOS	To analyze the association between PCOS and gynecological cancers, namely endometrial, breast, and ovarian cancer	There's higher risk of endometrial cancer in PCOS cohort. No association was observed between PCOS and ovarian or breast cancer
Harris and Terry [1]	Fertility Research and Practice	Systematic review	2016	11 individual studies and three meta-analyses on the associations between PCOS and endometrial cancer, eight studies and one meta-analysis for ovarian cancer, and 10 studies and one meta-analysis for breast cancer	To determine association between PCOS and endometrial, ovarian, and breast cancers	Women with PCOS were at a higher risk for endometrial cancer. A potentially increased risk of the borderline serous subtype of ovarian cancer is reported by two studies. No consistent association between PCOS and breast cancer observed
Gottschau et al. [21]	Gynecologic Oncology	Cohort study	2015	12,070 with PCOS	To explore the association between polycystic ovary syndrome (PCOS) and cancer, especially of the endometrium, breast, and ovary	Women with PCOS are at increased risk for endometrial cancer, whereas their risks of breast and ovarian cancer are similar to those of women in the general population
Shen et al. [20]	The Oncologist	Retrospective cohort study	2015	3,566 with PCOS, 14,264 matched patients without PCOS	To investigate whether PCOS raises the risk of developing uterine, ovarian, or breast cancer	PCOS might increase the risk of subsequent newly diagnosed uterine cancer
Prakansamut et al. [19]	Journal of The Medical Association Thailand	Cross-sectional study	2014	52 PCOS patients with abnormal menstrual pattern	To assess the occurrence of endometrial hyperplasia and endometrial cancer among PCOS patients with abnormal menstrual pattern	19.2% of patients with PCOS and abnormal menstrual pattern had endometrial hyperplasia or endometrial cancer
Barry et al. [12]	Human Reproduction Update	Systematic review and meta-analysis of observational studies	2014	11 studies for meta-analysis (919 women with PCOS and 72,054 non-PCOS controls)	To quantify separately the risk of endometrial cancer, ovarian cancer, and breast cancer in women with PCOS compared with non-PCOS controls, and quantify separately the risk to women of all ages as well as the risk to premenopausal women	Women with PCOS were at a significantly increased risk of endometrial cancer but the risk of ovarian and breast cancers was not significantly increased
Holm et al. [18]	Acta Obstetricia et Gynecologica Scandinavica	Retrospective observational trans-sectional study	2012	963 women with PCOS	To investigate the prevalence of endometrial hyperplasia and endometrial cancer in a well-characterized group of women with polycystic ovary syndrome and/or clinical/biochemical hyperandrogenism	The prevalence of endometrial cancer in women with polycystic ovary syndrome and/or clinical/biochemical hyperandrogenism is similar to that of the general population
Haoula et al. [14]	Human Reproduction	Systematic review	2012	14 studies, out of which five comparative studies were used for meta-analysis, 4,605 women which included 88 with PCOS	To determine the exact strength of the association between polycystic ovary syndrome (PCOS) and endometrial cancer (EC)	Women with PCOS are about three times more likely to develop EC compared with women without it

## Conclusions

The mechanisms contributing to gynecological cancers in females with PCOS are complex and still require exhaustive research in the form of large-scale and long-term studies to consolidate the association between them. After reviewing the 10 studies, it is evident that there is a significantly increased risk of endometrial cancer in women with PCOS. Though, a few studies have pointed out the limitations of lack of availability of data regarding other factors associated with cancer, such as smoking, alcohol consumption, and family history. Also, risk factors, such as high BMI, metabolic syndrome, and fertility, were unmatched in the majority of the studies. The risk of breast cancer and ovarian cancer is not significantly greater in women with PCOS as compared to those without it. A recent study has reported a reduced risk of ovarian cancer in women with PCOS, most reduction is seen in the endometrioid histotype, this could be because of the standardized use of oral contraceptives in the treatment of a majority of women with PCOS. A prospective cohort study assessing the association of PCOS and subtypes of endometrial, ovarian, and breast cancer is imperative in the future. This will not only aid in the consideration of the different confounding factors and mediators linked to cancer in women with PCOS, but it will also encourage clinicians to adopt a patient-specific approach.
